# Effects of nitrogen application on winter wheat growth, water use, and yield under different shallow groundwater depths

**DOI:** 10.3389/fpls.2023.1114611

**Published:** 2023-03-07

**Authors:** Yingjun She, Ping Li, Xuebin Qi, Shafeeq Ur Rahman, Wei Guo

**Affiliations:** ^1^ Farmland Irrigation Research Institute, Chinese Academy of Agricultural Sciences, Xinxiang, Henan, China; ^2^ Graduate School of Chinese Academy of Agricultural Sciences, Beijing, China; ^3^ Water Environment Factor Risk Assessment Laboratory of Agricultural Products Quality and Safety, Ministry of Agriculture and Rural Affairs, Xinxiang, Henan, China; ^4^ School of Environment and Civil Engineering, Dongguan University of Technology, Dongguan, Guangdong, China; ^5^ Ministry of Education (MOE) Laboratory for Earth Surface Processes, College of Urban and Environmental Sciences, Peking University, Beijing, China

**Keywords:** leaf area index, velocity of groundwater consumption, grain yield, water use efficiency, principal component analysis

## Abstract

Shallow groundwater plays a vital role in physiology morphological attributes, water use, and yield production of winter wheat, but little is known of its interaction with nitrogen (N) application. We aimed to explore the effects of N fertilization rate and shallow groundwater table depth (WTD) on winter wheat growth attributes, yield, and water use. Experiments were carried out in micro-lysimeters at WTD of 0.6, 0.9, 1.2, and 1.5 m with 0, 150, 240, and 300 kg/ha N application levels for the winter wheat (*Triticum aestivum* L.). The results showed that there was an optimum groundwater table depth (Op-wtd), in which the growth attributes, groundwater consumption (GC), yield, and water use efficiency (WUE) under each N application rate were maximum, and the Op-wtd decreased with the increase in N application. The Op-wtd corresponding to the higher velocity of groundwater consumption (Gv) appeared at the late jointing stage, which was significantly higher than other WTD treatments under the same N fertilization. WTD significantly affected the Gv during the seeding to the regreening stage and maturity stage; the interaction of N application, WTD, and N application was significant from the jointing to the filling stage. The GC, leaf area index (LAI), and yield increased with an increase of N application at 0.6–0.9-m depth—for example, the yield and the WUE of the NF300 treatment with 0.6-m depth were significantly higher than those of the NF150–NF240 treatment at 20.51%, and 14.81%, respectively. At 1.2–1.5-m depth, the N application amount exceeding 150–240 kg/ha was not conducive to wheat growth, groundwater use, grain yield, and WUE. The yield and the WUE of 150-kg/ha treatment were 15.02% and 10.67% higher than those of 240–300-kg/ha treatment at 1.2-m depth significantly. The optimum N application rate corresponding to yield indicated a tendency to decrease with the WTD increase. Considering the winter wheat growth attributes, GC, yield, and WUE, application of 150–240 kg/ha N was recommended in our experiment.

## 1 Introduction

Groundwater is essential for human beings and agricultural development, with more than 43% of irrigation water coming from groundwater (Döll et al., 2012; Russo and Lall, 2017; Jiang et al., 2020; Lian et al., 2022). Shallow groundwater is particularly the main component of the regional water cycle and one of the important sources of crop water, playing a significant role in sustainable agricultural production (Babajimopoulos et al., 2007; Liu et al., 2016). Shallow groundwater exists in many regions (Babajimopoulos et al., 2007), such as arid and semi-arid regions, the Yellow River basin in North China Plain, farmlands near irrigation districts, *etc*. (Wang et al., 2009; Zhu et al., 2018; Lai et al., 2022; Wang et al., 2022). Soil capillary voids and crop roots absorb shallow groundwater to replenish crops and dissipate into the atmosphere, which affects crop growth and yield (Liu et al., 2016; Soylu and Bras, 2022). However, in some sites (adverse groundwater table), it may bring about negative agricultural and environmental effects (Ibrahimi et al., 2013; Wang et al., 2016a). Wheat is an important grain crop worldwide and the second primary grain crop, with its sowing area accounting for approximately 20% in China (Liu et al., 2016; Zheng et al., 2020). Therefore, it is of great significance to study shallow groundwater for winter wheat growth, groundwater utilization, and yield formation.

As a significant water source for agricultural production, shallow groundwater utilization and crop water consumption characteristics have attained much attention. However, the effects of shallow groundwater on crop growth, water use, and yield are affected by many external factors, such as the depth of groundwater, irrigation precipitation, crop types, salt contents, *etc*. (Kahlown et al., 2005; Babajimopoulos et al., 2007; Ghamarnia and Daichin, 2015; Wang et al., 2016a). As for these external factors, numerous research have been done. Kahlown et al. (2005) used lysimeters to control different groundwater table depths (WTD) (0.5–3 m) and found that 90% and 80% of water consumption of wheat and sunflower at 0.5-m depth came from groundwater, but sugarcane, berseem, and sorghum could not survive at this WTD; the wheat yield was maximum at 1.5-m depth. This may be due to too shallow or too deep WTD that is not conducive to crop growth. For too shallow groundwater depth, the hypoxic or anaerobic environment in the root zone is not conducive to nutrient release and would lead to soil nutrient loss (Barrett-Lennard and Shabala, 2013; Najeeb et al., 2015; Ghobadi et al., 2017; Deng et al., 2021; Li et al., 2021; Langan et al., 2022); soil water deficit induces drought stress easily because of too deep groundwater depth, which is also unfavorable to crop growth (Xu et al., 2013; Han et al., 2015; Liu et al., 2017; Zhang et al., 2019). In addition, Zhu et al. (2018) found that the contribution rate of groundwater consumption (GC) to winter wheat evapotranspiration (ET_a_) in dry (2.72-m depth), normal (1.45-m depth), and wet seasons (1.49-m depth) was 58%, 47%, and 69%, respectively. Wang et al. (2016)a showed that the daily GC velocity (Gv) of silt loam over sandy loam was higher than that of sandy soil at the same WTD (1.5–3.5-m depth); irrigation and precipitation decreased the Gv (Yang et al., 2000; Wang et al., 2016a). The yield of deficient irrigation at shallow WTD (1.1–2.7-m depth) did not decrease significantly (Gao et al., 2017), while the increase of WTD and salinity reduced the groundwater contribution, seed oil yield, and water use efficiency (WUE) (0.6–1.1-m depth) under full irrigation (Ghamarnia and Daichin, 2015). In brief, concerning crop water use and yield, the existing research mostly focused on exploring the depth of groundwater and the combination of irrigation and salt content of different WTDs. However, chemical fertilizer is an indispensable nutrient for agricultural production, especially nitrogen (N) application, which is an essential component in promoting crop growth and yield formation (Zhang et al., 2011; Chen et al., 2014; Kumar et al., 2022). In order to maintain high crop yield and reduce the increasingly severe water shortage problem, the coupling of N and water application has always been a hot topic (Li et al., 2021; Javed et al., 2022). Nevertheless, it is surprising, under the condition of shallow groundwater depth, that whether N application rate affects crop growth, groundwater use, and yield formation is rarely reported.

Rational N application rate is beneficial for coordinating soil C/N ratio, improving soil organic C/N composition and other physicochemical properties, promoting crop and soil microbial growth, and increasing soil fertility and crop yield (Zhang et al., 2017; Deng et al., 2020; Liu and Li, 2022). However, improper N application, especially excessive N application, is not conducive to harmonizing soil nutrient composition (Qiu et al., 2016; Ren et al., 2019), reducing soil texture and greatly increasing nitrate residue, decreasing N use efficiency in farmlands (Treseder, 2008; He et al., 2018; Zhang et al., 2018; Ren et al., 2019; Wu et al., 2021), and ultimately limiting crop growth and yield (Yan et al., 2015). Excessive N application would also produce a series of environmental problems, such as greenhouse gas emissions, soil acidification, and groundwater nitrate pollution (Guo et al., 2010; Cui et al., 2018; Hu et al., 2018; Yu et al., 2019), so rational N application is significant for promoting sustainable agricultural development and environmental protection. The previous research mainly explored the rational N application interval from the aboveground irrigation. Zhou et al. (2019) found that the dry matter accumulation and yield of winter wheat under border irrigation with 240 kg N ha^-1^ were optimal. Si et al. (2020) showed that drip irrigation with N application of more than 240 kg N ha^-1^ was not conducive to winter wheat growth and water use. Kumar et al. (2022) reported that cotton N application of 225 kg N ha^-1^ with 600-mm irrigation resulted in better crop growth and yield in hot and arid areas; 210–270 kg N ha^-1^ with 140–215-mm irrigation was an efficient management mode, which could significantly increase the yield as reported in the study of Ji et al. (2014) and Sun et al. (2018). Huang et al. (2018) recommended 190 kg N ha^-1^ for winter wheat and 150 kg N ha^-1^ for summer maize based on agricultural production and environmental effects. Water and nitrogen migrate downward depending on gravity for crop absorption and utilization under surface irrigation and N application conditions, while in the shallow groundwater table area, groundwater migrates upward through soil and crop root absorption, and N application is applied from the soil surface. How do the groundwater and nitrogen affect crop growth, groundwater consumption, and yield then? The responses of crop growth, groundwater consumption, and yield to various combinations of groundwater depth and N application rates are unclear (Li et al., 2015; Zhang et al., 2018). Therefore, for sustainable agricultural development and agroecological protection, it is necessary to explore the effects of N application on crop growth, groundwater consumption, and yield formation under shallow groundwater table depths. Therefore, the objectives of this study were as follows: (1) to explore the effects of groundwater depth with N application rate on winter wheat growth attributes, yield, and water use; (2) to evaluate the function of groundwater depth and N application on the groundwater consumption characteristics of winter wheat; and (3) to obtain the optimal N fertilization and groundwater depth on the area of the shallow groundwater table.

## 2 Materials and methods

### 2.1 Experimental site

**Figure 1:**
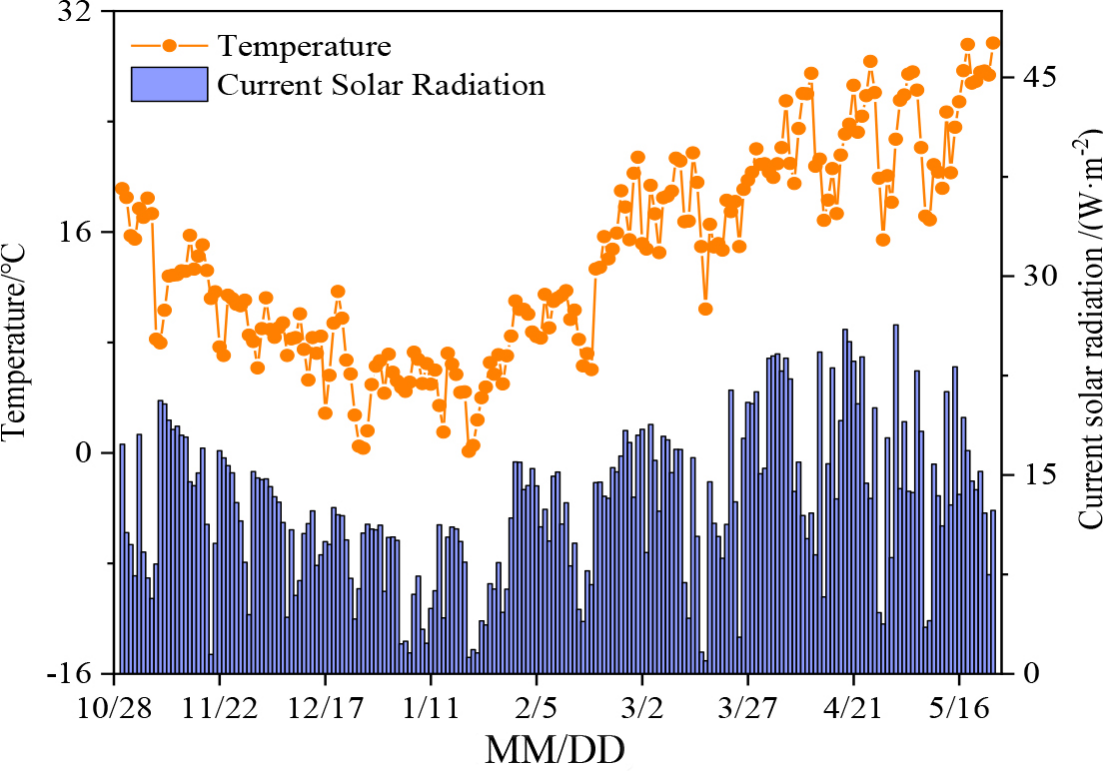
Daily temperature and solar radiation.

The experimental site is located at the Agricultural Water and Soil Environment Field Scientific Observation and Experiment Station (35°27′ N, 113°53′ E, and 73.2 m altitude) of the Chinese Academy of Agricultural Sciences in Xinxiang, Henan Province. The local climate is continental and monsoonal, with an average rainfall of 588.8 mm. The mean annual temperature and the potential evaporation were reported as 14.1°C and 2,000 mm, respectively. Meteorological data were obtained from a standard automatic weather station located in the experimental station (**Figure 1**).

A lysimeter was used to control the groundwater table depth and plant winter wheat. The diameter of the lysimeter was 40 cm, the height was 1.0–1.9 m, the side had soil extraction holes, and the inside was equipped with soil moisture monitoring probes and soil solution extractors. The details of the lysimeter were described by She et al. (2022). The water level scale on the Mariotte bottle was used to record the groundwater consumption. The water level was recorded at 8 a.m. every day. An electric awning was installed above the test area. The awning would be closed before raining or snowing to isolate the influence of precipitation. During the whole experiment, the test was not disturbed by precipitation.

The test soil was taken from the local farmland in four parts: 0–20 cm, 20–40 cm, 40–60 cm, and below 60 cm. After the back filter material was filled in the bottom of the lysimeter, the naturally air-dried soil was passed through a 5-mm sieve and backfilled in the order below 60 cm, 40–60 cm, 20–40 cm, and 0–20 cm. The backfilling bulk density was 1.40 g/cm^3^, and the soil was packed in layers every 2 cm. The soil was silt loam, and the other physicochemical properties are shown in **Table 1**.

**Table 1:** Physicochemical properties of the experimental soil.

Soil layer (cm)	EC us/cm)	OM (g·kg^-1^)	AN (mg·kg^-1^)	AK (mg·kg^-1^)	TN (g·kg^-1^)	TP (g·kg^-1^)	Soil granulometry composition		
							Clay (%)	Silt (%)	Sand (%)
0–20	270.00	12.29	17.27	128.33	0.85	0.63	18.26	47.43	34.31
20–40	313.33	9.87	13.30	81.33	1.25	0.59	18.09	45.93	35.97
40–60	364.00	8.78	7.93	81.67	1.52	0.53	17.84	44.04	38.78
>60	421.67	8.77	6.18	76.33	1.47	0.48	15.88	43.87	40.00

EC, electrical conductivity of soil; OM, organic matter of soil; AN, alkaline nitrogen of soil; AK, available potassium; TN, total nitrogen of soil; TP, total phosphorus of soil.

### 2.2 Experimental design

Groundwater table depth and nitrogen (N) fertilization were two factors in the experiment; the thickness of the vadose zone was based on the main root distribution of winter wheat in 0–0.6 m and simulated the surface, subsurface, and deeper subsurface soils (Liu and Luo, 2011; Liu et al., 2015; Xin et al., 2019). The groundwater depth was set as 0.6 m (G1), 0.9 m (G2), 1.2 m (G3), and 1.5 m (G4), respectively. The N application rates were 0 kg/ha (0 g/lys., lysimeter) (NF 0), 150 kg/ha (1.88 g/lys.) (NF150), 240 kg/ha (3.01 g/lys.) (NF240), and 300 kg/ha (3.77 g/lys.) (NF300), respectively. The experiment adopted a completely random block design with 16 treatments, including three replications and 48 lysimeters in total.

The winter wheat (*Triticum Aestivum L.*) was sown on November 1, 2021, with the “Bainong 4199” cultivar. The seeding amount was approximately the local planting habit of 225 kg/ha (60 grains/lsi.), and the sowing depth was 5 cm. Wheat was harvested on May 21, 2022, with a total growth of 202 days. N, P, and K fertilizers were applied using common urea (46% N), calcium magnesium phosphate (12% P_2_O_5_), and potassium sulfate (50% K_2_O). According to the local field application rate, 150 kg/ha P (1.88 g/lys.) and 120 kg/ha K (1.51 g/lys.) were applied, respectively. N fertilizer was applied in the form of base fertilizer and topdressing with a ratio of 6:4, with a mixture of 60% N, 100% P, and 100% K fertilizer applied before seeding, and 40% N fertilizer of topdressing was applied at the jointing stage. In the experiment, a canopy was used to avoid rainfall interference, and irrigation was carried out based on the soil moisture from the probe in 0–40 cm and the crop growth status. Meanwhile, irrigation was controlled appropriately to highlight the role of groundwater in the experiment. The irrigation period and the irrigation amount are shown in **Table 2**, and the irrigation amount of each lysimeter was consistent. Spraying of pesticides was also done in time to prevent winter wheat pests and diseases.

**Table 2:** Irrigation period and irrigation amount of winter wheat.

Irrigation stage	Irrigation amount (mm/lys.)	Irrigation stage	Irrigation amount (mm/lys.)
Seeding	55.73	Jointing	23.89
Over-wintering	31.85	Booting	15.92
Regreening	19.90	Filling	47.77

The seeding stage of winter wheat means the period of time from the sowing to the over-wintering stage; the same is true below.

### 2.3 Methods for investigation

#### 2.3.1 Growth attributes

Three representative plants were randomly selected to measure the crop height and the green leaf area. Before the heading stage, the crop height was measured from the base of the wheat to the highest point of the leaf with a ruler; in the heading to maturity stage, the base of the wheat to the top of the ear (without awn) was measured as the crop height. The measured stages included the regreening, jointing, heading, anthesis, filling, and maturity stages. The length and the width of the leaves were measured by the ruler method, and the leaf area index (LAI) was calculated by the coefficient method (0.83) in the jointing, anthesis, filling, and middle filling stages.

#### 2.3.2 Daily groundwater consumption velocity

The daily groundwater consumption velocity of winter wheat was calculated by the water amount change of the Mariotte bottle each day (Gv, mm/day). The groundwater consumption at each stage and during the whole growth period was accumulated *via* the daily water amount (GC, mm).

#### 2.3.3 Soil moisture

Soil moisture was obtained by combining oven drying method and online data acquisition. Actual evapotranspiration (ET_a_) was estimated using the soil water balanced equation as follows:


$$ET_{a}=P+I+GC-\left(D+\Delta W\right)$$

where ET_a_ is crop evapotranspiration, *P* is precipitation (mm), *I* is irrigation (mm), GC is groundwater consumption (mm), *D* is deep leakage loss, which is not generated due to controlled irrigation in the experiment (mm), and *ΔW* is the change of soil water storage in the vadose zone (mm). *P* and *D* were zero in the experiment.

#### 2.3.4 Grain yield

After harvest, the wheat needed for dry matter measurement and components of yield were removed from the lysimeter, and the remaining wheat was weighed to calculate the yield.

#### 2.3.5 Water use efficiency

The following formulas were used to calculate the water use efficiency and groundwater use efficiency:


$$WUE=\frac{Y}{ET_{a}}$$

$$GWUE=\frac{Y}{GC}$$

where WUE is water use efficiency (g·lys^-1^·mm^-1^), GWUE is groundwater use efficiency (g·lys^-1^·mm^-1^), and *Y* is grain yield (g·lys^-1^).

### 2.4 Statistical analysis

In SPSS 23.0 (SPSS Inc., Chicago, IL, USA), analysis of variance was used to distinguish the effects of different groundwater depth and N application rates on growth attributes, GC, yield, WUE, and GWUE of winter wheat. Duncan’s multiple-range test in the mode of the general model in SPSS 23.0 was used to compare the significant differences among different treatments (*p*< 0.05). Pearson’s correlation coefficients were used to analyze the relationships among growth attributes, GC, yield, and water use efficiency. The dimensionality reduction method was used to conduct principal component analysis and calculate the combined scores of different groundwater depth and N applications by a method from Huang et al. (2022) and Zheng et al. (2022). All figures were created using OriginPro 2021 (OriginLab Corporation, Northampton, MA, USA), and the bars in the figures represent the standard errors of the mean (Xu et al., 2018).

## 3 Results

### 3.1 Growth attributes

#### 3.1.1 Crop height

**Figure 2:**
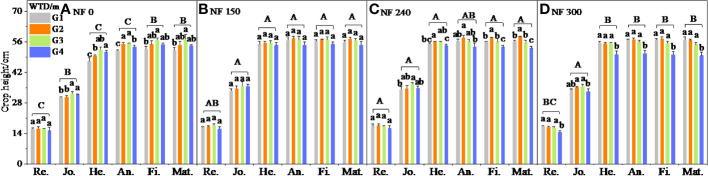
Winter wheat crop height at different growth stages. Re., Jo., He., An., Fi., and Mat. mean the regreening, jointing, heading, anthesis, filling, and maturity stage, respectively. Different lowercase letters indicate significant differences between different WTD under the same N application (P < 0.05), and uppercase letters indicate significant differences between different N application treatments (P < 0.05). The same is true below.

The WTD and N application significantly affected the crop height growth (**Table 3**). The crop height was not significantly different between WTD treatments at the regreening stage under NF0–NF240, while the G1–G3 treatment was significantly higher than the G4 treatment under NF300 (**Figure 2**). With the progress of growth, the crop height of the G3–G4 treatment was significantly higher than that of the G1–G2 treatment under NF0 (**Figure 2A**). Under NF150–NF240, the crop height increased first and then decreased with the WTD increase, and the G2–G3 treatments were significantly higher than those of G1 and G4 treatments under NF240 (**Figures 2B, C**); under NF300, the G1–G3 treatment was significantly higher than the G4 treatment (**Figure 2D**). The crop height of N application treatment from the regreening to jointing stage was significantly higher than that of the NF0 treatment; from the heading to maturity stage, the average crop height of NF150–NF240 treatments was 4.94% and 2.22% significantly higher than that of the NF0 and NF300 treatments, respectively (**Figure 2**), indicating that insufficient N application and excessive N application were both not conducive to the growth of winter wheat height.

**Table 3:** Two-way ANOVA of crop height, leaf area index (LAI), yield, and water utilization in winter wheat.

Factor	Crop height			LAI	Gv or GC			Yield	ET_a_	WUE	GWUE
	Re.-Jo.	He.	An.-Mat.	Jo.-MFi.	Se.-Re.	Jo.-Fi.	Mat.				
WTD	**	**	**	**	**	ns	**	*	**	**	**
NF	**	**	**	**	ns	**	ns	**	**	**	ns
NF * WTD	ns	**	**	**	ns	**	**	**	**	**	**

Re.-Jo., regreening to jointing stage; An.-Mat., anthesis to maturity stage; Jo.-MFi., jointing to the middle filling stage; Se.-Re., seeding to regreening stage; Jo.-Fi., jointing to the filling stage; Gv, daily velocity of groundwater consumption (GC).

*P< 0.05; **P< 0.01; ns, no significant difference between treatments.

#### 3.1.2 Leaf area index

**Figure 3:**
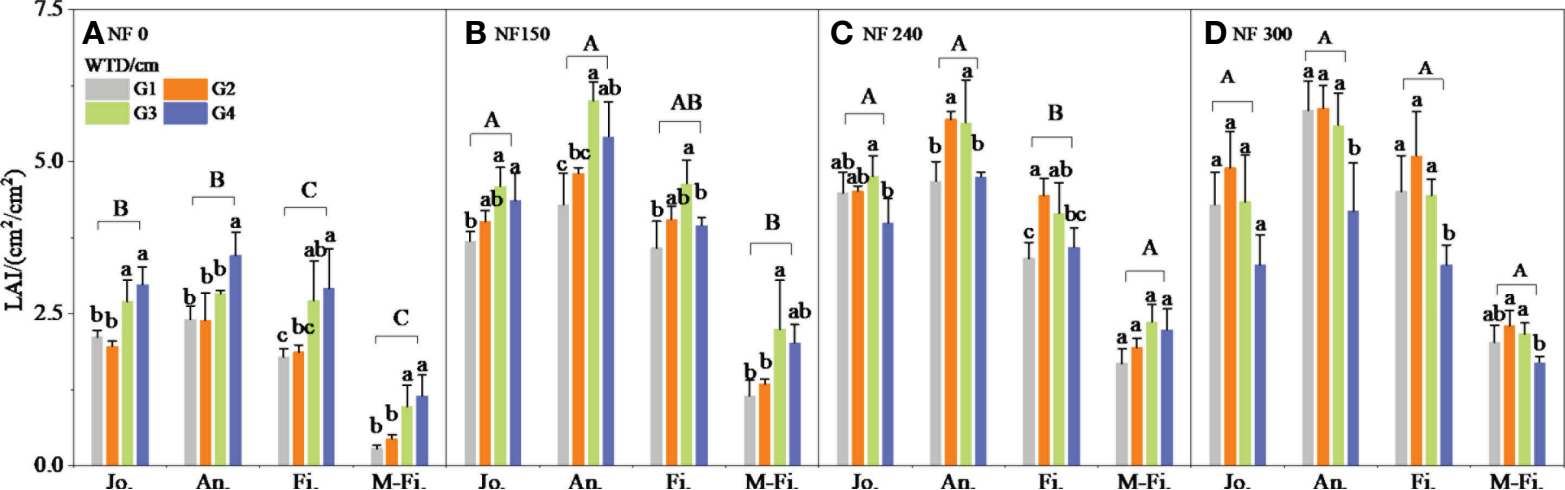
The LAI of winter wheat. M-Fi. means the middle filling stage, The same is true below. Different lowercase letters indicate significant differences between different WTD under the same N application (P < 0.05), and uppercase letters indicate significant differences between different N application treatments (P < 0.05).

The winter wheat LAI in the jointing to the middle of the filling stage increased with the WTD increase and was significantly higher in the G3–G4 treatment than in the G1–G2 treatment under NF0 (**Figure 3A**). The LAI of NF150-NF300 increased first and then decreased with the WTD increase, and the maximum values were NF150G3, NF240G2-G3, and NF300G2, respectively. Among them, the average LAI of G3 treatment increased significantly by 37.52% than that of G1 treatment under NF150; the LAI of G2–G3 treatment increased by 15.68%–16.64% from that of G1 treatment significantly under NF240 (except the maturity stage). The LAI of G1–G3 treatments was significantly higher than that of G4 treatments, with an average increase of 32.58%–45.56% under NF300 (**Figures 3B–D**).

The LAI of NF300 treatment was significantly higher than that of NF150-NF240 treatment, which increased by 6.98% and 11.24% in the filling stage. The NF240-NF300 treatment was significantly higher than the NF150 treatment, which was 21.73% and 21.26% higher in the middle stage of filling (**Figure 3**), indicating that increasing the N fertilizer application at 0.6–1.5-m depth could promote leaf growth and delay leaf yellowing to some extent.

### 3.2 Groundwater utilization of winter wheat

#### 3.2.1 Daily groundwater consumption velocity

**Figure 4:**
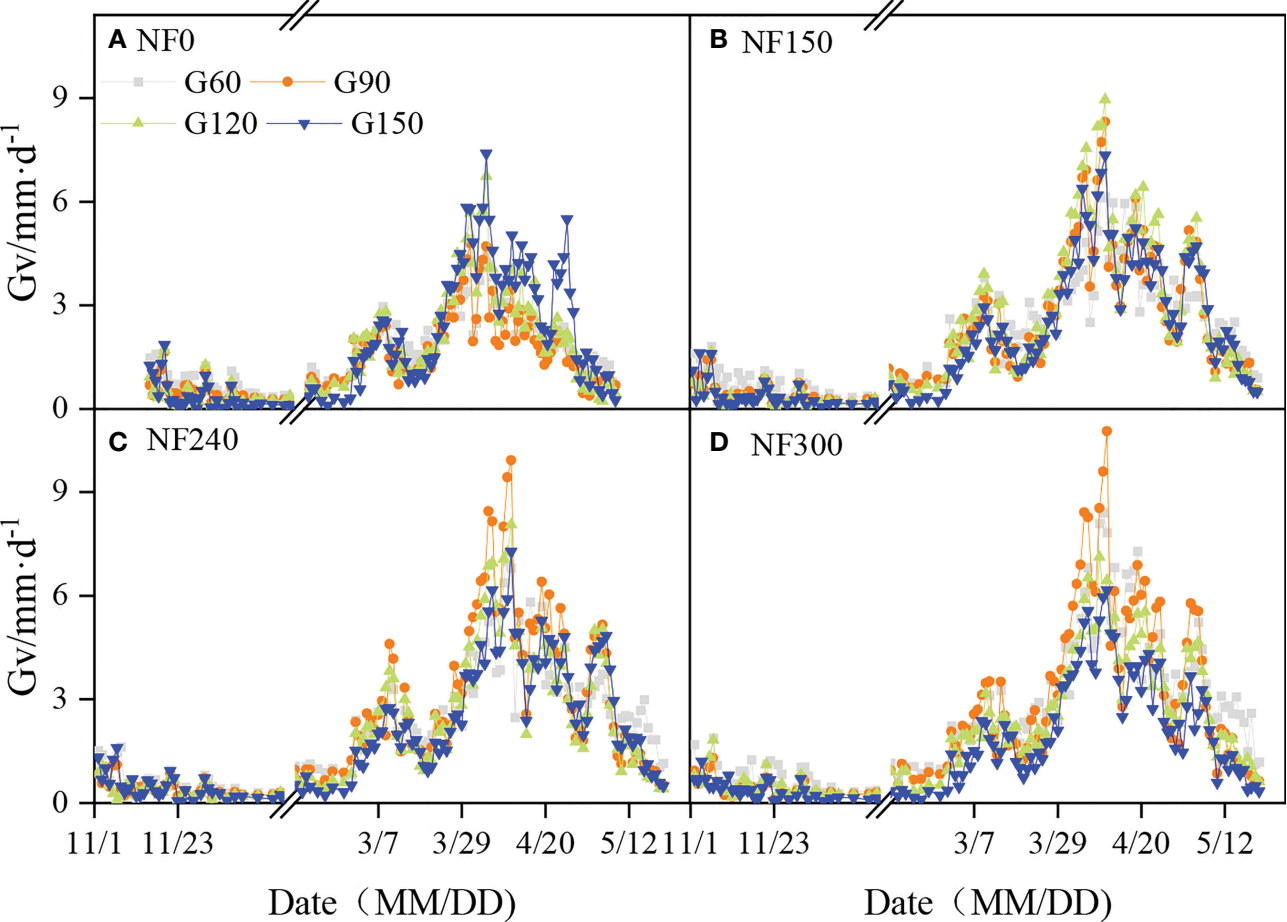
Daily groundwater consumption velocity of winter wheat. MM/DD means month and days.

After sowing, the daily groundwater consumption velocity (Gv) gradually decreased and maintained a low level (0.33 mm/day), with almost no consumption (0.17 mm/day) at the over-wintering stage. The Gv began to rise in the regreening stage, appeared at the first peak in the jointing stage, and reached the maximum in the booting to anthesis stage. Two peak values emerged at the filling stage, and a continuous decrease was shown at the later filling to maturity stage (**Figure 4**).

The Gv decreased with the WTD increase at the seeding to regreening stage and maturity stage, and the Gv of G1 treatment was significantly higher than that of G4 treatment under the same N application (**Supplementary Figure S1**). At the jointing to filling stage, the WTDs corresponding to the maximum Gv (optimal groundwater table depth, Op-wtd) in each N application were NF0G4, NF150G3, NF240G2, and NF300G1-G2, respectively. The Gv of Op-wtd treatments began to increase rapidly at the late jointing stage significantly (March 28–30). The Gv of the G4 treatment under NF0 application was significantly higher than that in the G1–G3 treatment at the booting to the filling stage (**Supplementary Figure S1A**) (**Figure 4A**). The Gv for the Op-wtds of the NF150-NF300 application was significantly higher than that of other WTD treatments at the heading stage (**Supplementary Figures S1B–D**).

Nitrogen application significantly affected the Gv of winter wheat (**Supplementary Figure S2**; **Figure 4**; **Table 3**). At the jointing to maturity stage, the Gv showed the order NF300 > NF150-NF240 > NF0 (*p*< 0.05) in G1 depth (except the jointing stage) and the order NF240-NF300 > NF150 > NF0 in G2 depth (except the maturity stage) (*p*< 0.05); little difference existed in Gv values among all N application treatments in the G3–G4 depth, but the highest Gv appeared in the NF150 treatment.

#### 3.2.2 Groundwater consumption of winter wheat

**Figure 5:**
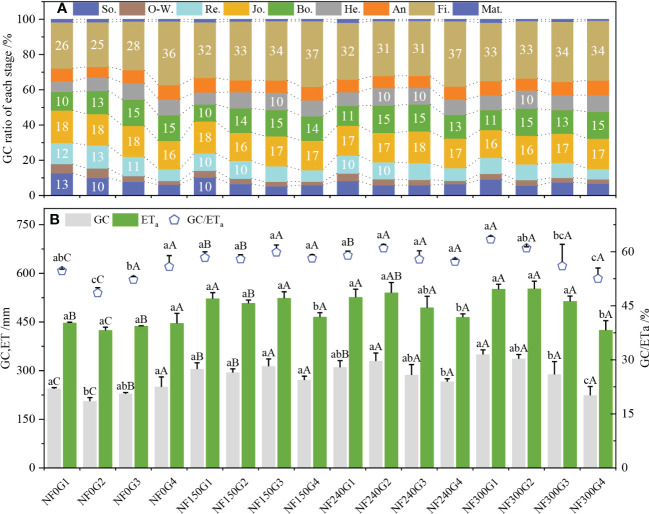
Proportion of GC of each stage, GC, ETa, and GC/Eta. Se., O-W., Re., Jo., Bo., He., An., Fi. and Mat. mean seeding, over-wintering, regreening, jointing, booting, heading, anthesis, filling and maturity stage, respectively. Different lowercase letters indicate significant differences were among different WTDs under the same N application rate, P < 0.05. Capital letters indicate significant differences among different N application rates under the same WTD, P < 0.05. The same is true below.

The GC-Fi. accounted for the largest proportion in the total GC, which was 26%–37% and tended to increase with the WTD increase, and the difference was obvious in NF0. The proportion of GC-Fi. of the NF150-NF300 treatment was higher than that of the NF0 treatment under the G1–G2 depth. Compared with the G3–G4 treatment, the G1–G2 treatment consumed more groundwater at the booting stage and before, which was 49.37%–59.37%, but the difference became smaller with the increase in N application (**Figure 5A**).

The WTD (OP-wtd) corresponding to the maximum GC and GC/ET_a_ in the NF0, NF150, NF240, and NF300 groups was G4, G3, G2, and G1, respectively, and the OP-wtd showed a decreasing trend with the increase in N application (**Figure 5B**). The GC in the G1 and G4 treatments was significantly higher than that in the G2–G3 treatments under NF0 application. Under NF240 application, the GC in the G1–G3 treatment was significantly increased by 16.55%, 23.88%, and 7.65% than that in the G4 treatment, respectively. Under NF300 application, the average GC and GC/ET_a_ in the G1–G2 treatments significantly increased by 34.12% and 14.62% higher than those in the G3–G4 treatments.

The OP-wtd corresponding to the maximum ET_a_ of 150–300-kg/ha N application was G3, G2, and G2, respectively. ET_a_ in the G1–G3 treatment was significantly higher than that in the G4 treatment, with an average increase of 11.19%, 11.91%, and 26.94%, respectively (**Figure 5B**). At G1 depth, the GC and GC/ET_a_ increased with the increase in N application, which was NF300 > NF150-NF240 > NF0 (*P*< 0.05); at G2 depth, this was NF240-NF300 > NF150 > NF0 (*P*< 0.05). At G3 and G4 depth, the GC, ET_a_, and GC/ET_a_ increased first and then decreased with the increase in N application, and the maximum values were from the NF150 treatment.

### 3.3 Grain yield, WUE, and GWUE

**Figure 6:**
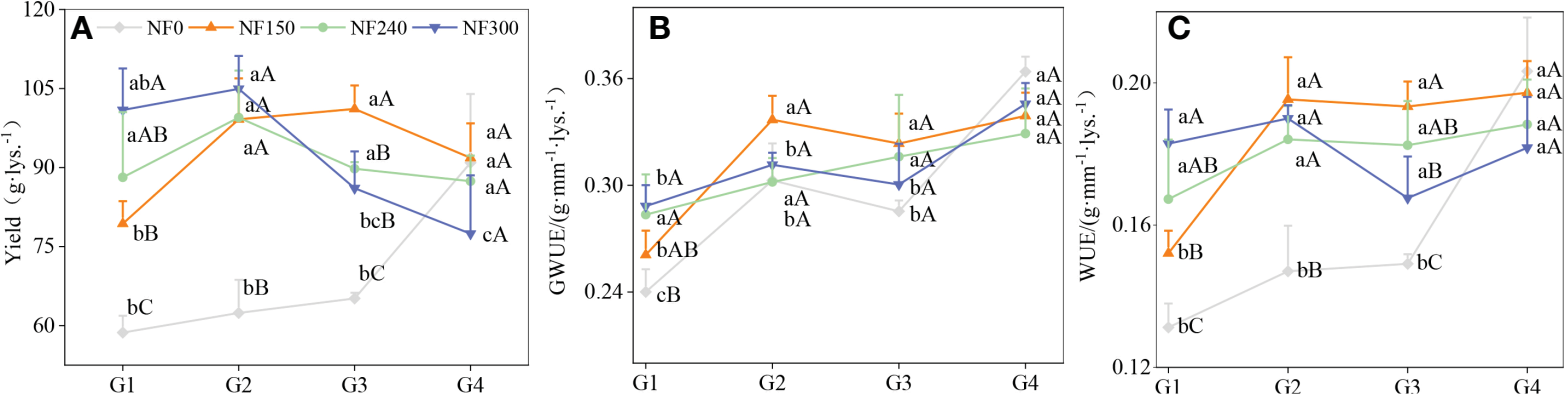
Winter wheat yield, WUE and GWUE. Different lowercase letters indicate significant differences were among different WTDs under the same N application rate, P < 0.05. Capital letters indicate significant differences among different N application rates under the same WTD, P < 0.05.

The yield and WUE of NF0 increased with the WTD increase. The yield, WUE, and GWUE of the G4 treatment were significantly higher than those of the G1–G3 treatment. The yield increased first and then decreased with WTD under the NF150-NF300 application; the WTD corresponding to the maximum yield was G3, G2, and G2, respectively. Under the NF150 application, the yield, WUE, and GWUE of the G2–G4 treatment were significantly higher than those of the G1 treatment, with an average increase of 22.73%, 28.43%, and 27.71%, respectively. Under the NF300 application, the yield of the G1–G2 treatment was significantly higher than that of the G3–G4 treatment, with an average increase of 25.89%, but WUE was significantly lower than that of the G4 treatment (**Figure 6**).

Under G1 depth, the yield, WUE, and GWUE increased with increase in N application, and the yield and WUE of the NF300 treatment were 20.51% and 14.81% higher, on average, compared with the NF150-NF240 treatment. The yield and WUE of G2 depth under the NF150-NF300 treatment were significantly higher than those of the NF0 treatment. The yield and WUE of G3 depth were as follows: NF150 > NF240-NF300 > NF0 (*P*< 0.05), and the yield and WUE of NF150 treatment were 15.02% and 10.67% higher compared with the NF240-NF300 treatment (**Figure 6**). N application (*p*< 0.01) had a stronger effect on yield than groundwater depth (*p*< 0.05) (**Table 3**). For G1–G2 depth, increased N application could promote the yield increase effect of groundwater, and NF150 showed an obvious positive yield increase effect. However, when the WTD > G2 and the N application rate >150 kg/ha, increasing the N application would aggravate the effect of groundwater depth on yield reduction. The yield of NF300G1, NF300G2, NF240G2, and NF150G3 was significantly higher than that of other combined treatments. The Op-wtd corresponding to the highest yield tended to reduce with the increase in N application.

### 3.4 Analysis by synthesis

#### 3.4.1 Fitting analysis of optimal N application rate based on yield

**Figure 7:**
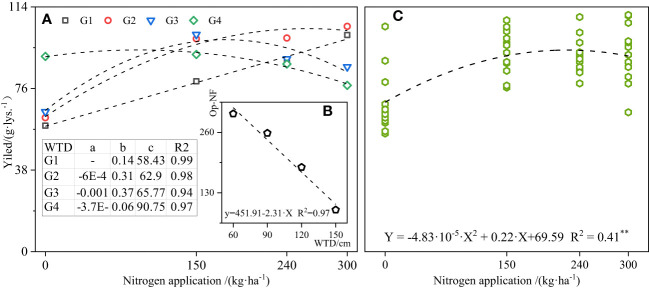
Fitting analysis of appropriate N application rate based on yield. In **(A)**, a is a quadratic coefficient, b is a linear coefficient, c is a constant term, and ** means P < 0.01.

Quadratic relationships between wheat yield and N application rate under the same WTDs (the linear relationship at G1 depth) (*R* ^2^ = 0.94–0.99) are shown in **Figure 7A**. The appropriate N application rate at the highest yield under each WTD could be obtained, and this N application rate had a decreasing trend with the WTD increase (**Figure 7B**). In the range of 0.6–1.5-m depth, the yield and N application rate showed a significant quadratic relationship (**Figure 7C**), and the N application rate was 227.74 kg/ha, the yield reached 94.64 g/lys, corresponding to the WTD of 96.96 cm.

#### 3.4.2 Person’s correlation analysis

**Figure 8:**
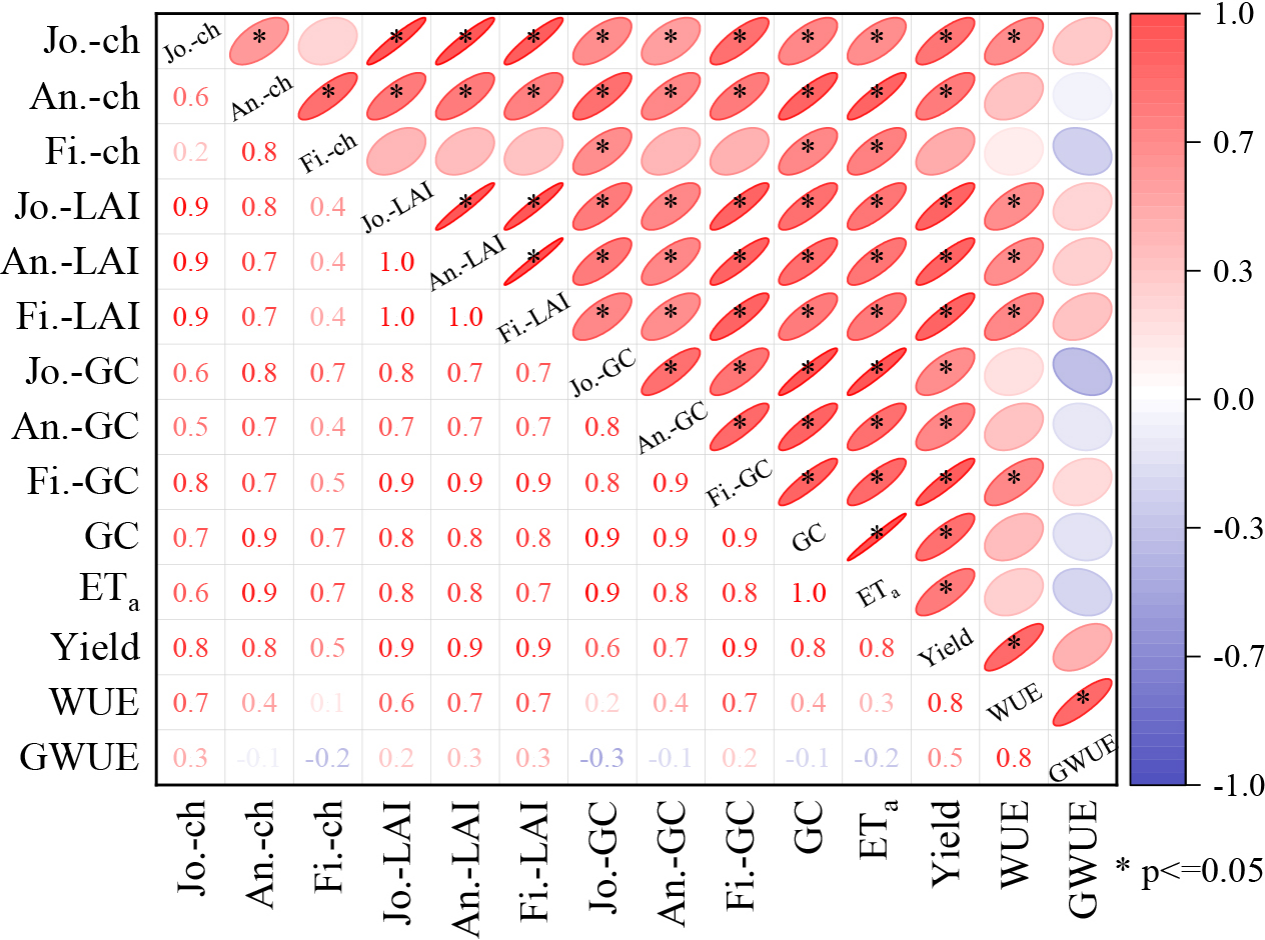
Correlation thermogram.

Changes in crop height and leaf area were significantly correlated with yield and water utilization (**Figure 8**). Crop height at the jointing and anthesis stage was positively correlated with LAI, GC, and yield. LAI was positively correlated with GC, ET_a_, yield, and WUE. GC was positively correlated with yield.

#### 3.4.3 Multiple regression analysis

Multiple stepwise regression analysis was conducted to select the growth attributes and water contribution indexes that had the greatest impact on yield and water utilization. The results showed that X4, X8, X9, X10, Y1, Y4, Y5, and Y8 significantly affected yield and water use efficiency (**Table 4**).

**Table 4:** Multiple regression equations of groundwater consumption (GC), yield, and water use efficiency (WUE) on growth and water utilization indicators.

Indicators	X	Y
Yield	*Z* = 13.50·X9 + 37.15 (R^2 =^ 0.80**)	*Z* = -0.49·Y4 + 0.82·Y5 + 0.63·Y8+21.21 (*R* ^2^ = 0.95**)
WUE	*Z* = 0.022·X10 + 0.14 (R^2 =^ 0.45**)	*Z* = -0.002·Y1 - 0.001·Y4 + 0.001·Y8+0.17 (*R* ^2^ = 0.88**)
GC	Z = 14.43·X4 + 12.86·X8-576.92 (*R* ^2^ = 0.80**)	—

The growth indicators at each growth stage were selected as the first part of independent variables (Part I). X1 - X10 were crop height at the regreening, jointing, heading, anthesis, filling, and maturity stage, and LAI at the jointing, anthesis, filling, and midfilling stage, respectively. The GC index of each stage was selected as the second part of the independent variables (Part II). Y1-Y9 were GC at the seeding, over-wintering, regreening, jointing, booting, heading, anthesis, filling, and maturity stages, respectively. GC, yield, and WUE were selected as the dependent variables Z_GC_, Z_yield_, and Z_wue_. ** means p<0.01.

#### 3.4.4 The comprehensive evaluation score

**Figure 9:**
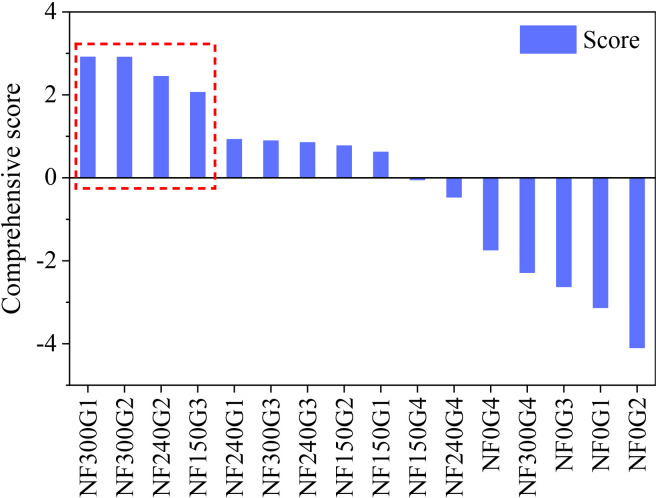
Scores and ranking of combined treatment of WTD with N application.

Crop growth attributes and water use parameters selected from **Table 4** were combined with yield, GC, ET_a_, WUE, and GWUE to constitute the score items. The comprehensive evaluation score function of WTD combination with N application treatments was constructed by the variance contribution rate of each principal component as the weight: *S* = 0.732·S1 + 0.267·S2, and the score and the ranking of each combination treatment were obtained (**Figure 9**). The top four combined treatments with comprehensive scores were NF300G1, NF300G2, NF240G2, and NF150G3 (**Figure 9**). These four optimal combined treatments were obtained by synthesizing the better growth attributes, water use, and yield indexes of winter wheat. More importantly, from the four combination treatments with the highest combined scores, the optimal N application rate decreased with the increase of groundwater depth, or the optimal groundwater depth reduced with the increase of N application rate.

## 4 Discussion

As one of the main water sources of crops, groundwater significantly affects crop growth and yield formation. The previous study has pointed out that crop groundwater consumption (GC) decreased with the WTD increase (Yang et al., 2011). Still another study showed that the shallow WTD’s contribution to crop root zone water was not necessarily greater. The optimal groundwater table depth (Op-wtd) with the best root growth and water consumption was existent (Gou et al., 2020). Because the influence of groundwater depth on crop growth attributes and yield was generally reflected in the balance between crop water absorption and crop growth environment (Zhang et al., 2018), too deep and too shallow groundwater were both not conducive to crop growth (Kahlown et al., 2005; Kang et al., 2007; Liu et al., 2014; Kadioglu et al., 2019; Zhang et al., 2019), but the Op-wtd was influenced by soil salinity, crop type, soil structure, agricultural practices, *etc*. (Babajimopoulos et al., 2007; Liu et al., 2015; Barbeta and Penuelas, 2017; Wang et al., 2018). The growth, water use, and yield of winter wheat in our research were obviously affected by N fertilization under shallow groundwater depth.

Water consumption at the beginning and at the end of crop growth was dominated by soil evaporation, and the depth of groundwater was the significant factor affecting the intensity of groundwater consumption. From the seedling stage to the regreening stage and the maturity stage, the groundwater consumption of winter wheat decreased with the increase of WTD (**Supplementary Figure S1**; **Table 3**). From the jointing to filling stage, with winter wheat growth and air temperature rising (**Figures 1** –**3**), the groundwater consumption increased rapidly (**Figure 4**). The groundwater consumption at these stages was closely related to the crop growth environment and water and fertilizer supply conditions. The optimal groundwater depths appeared for the optimal growth attributes, water utilization, and yield under the N application groups (**Figures 3**, **4**, **6**). Soylu et al. (2014) showed that the 0.8–1.0-m depth was the critical depth for groundwater to produce anaerobic stress, so the growth of winter wheat may be inhibited (Gou et al., 2020; Deng et al., 2021). In our study, winter wheat growth attributes and groundwater consumption decreased significantly at 0.6–0.9-m depth with deficient N fertilization (**Supplementary Figures S2A, B**; **Figure 8**), which may be due to anaerobic stress caused by the high water level. However, increased N application at the depth of 0.6–0.9 m significantly promoted winter wheat growth, thus increasing groundwater consumption and yield (**Figure 4**; **Supplementary Figures S2A, B**; **Figure 8**). The crops’ water and fertilizer requirements were high during the jointing to the filling stage. The relatively thin vadose zone of 0.6–0.9 m (<1.0 m) can ensure sufficient groundwater supply for winter wheat. Nitrogen is a significant element for crop growth. Exogenous N fertilization increased the nitrogen uptake sources of the crop, and it could alleviate waterlogging stress and supplement nitrogen loss due to denitrification at high water levels to some extent (Li et al., 2021; Chlapecka et al., 2022; Zhang et al., 2022). Therefore, sufficient water and nitrogen jointly promoted winter wheat growth, water utilization, and yield formation at a depth of 0.6–0.9 m (**Figures 3**, **6**, **8**; **Supplementary Figures S2A, B**).

Adding N application could supplement soil N content to some extent, enhance the resilience of crops (Ma et al., 2019), and maintain normal crop growth and yield formation (**Figures 3**, **6**, **8**). However, higher N application was not always better. Especially superfluous N fertilization would significantly increase the nitrate residue in soil under the thick vadose zone with less rainfall or irrigation (Zhou et al., 2016). In our experiment, for the WTD of more than 1.2 m, the upward water refill path of groundwater was lengthened, the soil moisture in the upper vadose zone decreased, and the groundwater consumption by crops was reduced (**Figure 5B**). WTD may be deeper, and the upper soil moisture was too late to be replenished with limited irrigation (**Supplementary Figures S2C, D**). High N application rate (>150–240 kg/ha) significantly enlarged soil inorganic nitrogen content and deepened soil drought (Wang et al., 2016b; Zhang et al., 2020; She et al., 2022). Less water and more nitrogen in soil resulted in the imbalance of water and fertilizer supply for the crop, water stress limited the nitrogen availability (Gu et al., 2015), and a high N application rate inhibited crop growth and crop water absorption (**Figures 2B–D**, **3B–D**, **Supplementary Figures S2C, D**) (Rasmussen et al., 2015; Liu et al., 2018), resulting in yield reduction (**Figures 6**, **8**).

N application is an important factor promoting crop growth and yield formation, but excessive N application is not conducive to crop growth and yield formation, so appropriate N application has become the focus of attention of researchers and agricultural workers (Ren et al., 2019; Yu et al., 2019; Kumar et al., 2022). Previous studies showed that N application increased crop yield, but excessive N fertilization would lead to “diminishing returns” (Huang et al., 2018; Sun et al., 2018; Zhou et al., 2019; Si et al., 2020; Kumar et al., 2022). The optimal yield could be obtained by applying 227.74 N kg/ha in shallow groundwater depth (0.6–1.5 m), and the optimal N application tended to decrease with the WTD increase (**Figures 7**, **9**). Combined with actual production conditions, due to the large perennial amount of fertilization, most areas of China had high nitrate content in the soil vadose zone (Zhou et al., 2016; Liang et al., 2020). The groundwater table is deep, and the conventional N application of 300 kg/ha needs to be reduced (Cui et al., 2018; Si et al., 2020). The WUE and the GWUE under the deeper groundwater table (1.5 m) were significantly higher than those of the shallower groundwater table (0.6 m) treatment under 0–150 kg/ha N application, which was similar to the findings of Huo et al. (2012) and Liu and Luo (2011). However, there was no significant difference in N application between 240 and 300 kg/ha. This result was different from the research of Zhang et al. (2022), which may be due to soybean and cover crops that limited surface water evaporation to some extent in the study of Zhang et al. For N application in shallow groundwater depth areas, our results indicated that increasing the N application was beneficial to improve the WUE of crops at the high groundwater table, while reducing the N application could be appropriate at the low groundwater table. On the other hand, our results were obtained based on the lysimeter, and the water table controlled by the lysimeter was constant. Considering the complex and changeable meteorological and environmental conditions and the fluctuating water tables under natural conditions (Zheng et al., 2020), future long-term research is needed in the field.

It is obvious that the N application amount was another important factor affecting crop growth, water use, and yield besides the depth of groundwater in the shallow groundwater area (**Figures 3**, **6**; **Table 3**). It suggests that, in shallow groundwater areas, soil elements (such as nitrogen) play a vital role in crop growth and yield formation besides water’s apparent effects. However, this point has rarely been considered in previous studies. Therefore, the interaction mechanism of groundwater on crop physiological growth and related elements needs to be explored in shallow groundwater areas, such as the nitrogen absorption mechanism of crop roots at the groundwater to soil interface (G–S interface), the occurrence form of nitrogen at the G–S interface, its functional response to the environment, and its mechanism of action, of which need to be studied in depth.

## 5 Conclusion

The GC was mainly affected by groundwater depth which decreased with the increase in groundwater depth at the seeding to the regreening and maturity stages. From the jointing to filling stage, N application and the interaction between N application and groundwater depth had a significant effect on GC, and an optimal groundwater depth appeared under each N application group beginning from the late jointing stage.

N application could improve crops’ environmental resilience, mobilize groundwater utilization at 0.6–0.9-m depth, and promote crop growth and yield, while at >1.2-m depth, an increase in N application (>150–240 kg/ha) was not conducive to crop growth, and water use and the yield decreased significantly. The GC and GC/ET_a_ of the 300-kg/ha N treatment were significantly higher by 14.67% and 6.99% than those of the 150-kg/ha N treatment at 0.6–0.9-m depth. The yield and the WUE of the 300-kg/ha treatment at 0.6-m depth, respectively, were 20.51% and 14.81% significantly higher, on average, compared with the 150–240-kg/ha N treatment. At 1.2–1.5-m depth, the GC/ET_a_, GC, and ET_a_ were better under the 150-kg/ha N application. The yield and the WUE of the 150-kg/ha N treatment were 15.02% and 10.67% higher than those of the 240–300-kg/ha N treatment at 1.2-m depth.

In conclusion, for shallower groundwater depths (0.6–0.9 m), more N fertilization was beneficial to winter wheat growth, water use, and yield formation. However, for deeper groundwater depth (>1.2 m), a high N application rate (300 kg/ha) inhibited crop growth and was not conducive to water use and yield formation, which reduced the 20%–50% N application rate and was relatively beneficial. The effects of shallow groundwater on crop water use and yield are likely to imply nitrogen contribution. The interaction mechanism of groundwater on crop physiological growth and related elements needs to be explored in shallow groundwater areas. The nitrogen uptake mechanism of crop roots at the groundwater and soil interface (G–S interface), the occurrence form of nitrogen at the G-S interface, its functional response to the environment, and its mechanism of action should be deeply studied.

## Data availability statement

The original contributions presented in the study are included in the article/**Supplementary Material**. Further inquiries can be directed to the corresponding author.

## Author contributions

YS and XQ: conceptualization. YS, PL, WG, and SR: writing— original draft preparation. YS and XQ: writing—review and editing. PL and XQ: funding acquisition. All authors contributed to the article and approved the submitted version.
