# Supplementary material for: Effects of nitrogen application on winter wheat growth, water use, and yield under different shallow groundwater depths
Source: Front Plant Sci. 2023 Mar 7;14:1114611. doi: 10.3389/fpls.2023.1114611 (PMC10028210; doi:10.3389/fpls.2023.1114611)
Supplement: Supplementary file 1 [file DataSheet_1.docx]

*Effects of nitrogen application on winter wheat growth, water use and yield under different shallow groundwater depths*

Yingjun She^1,2^, Ping Li^1,3^, Xuebin Qi^1, 3*^, Shafeeq Ur Rahman^4,5^, Wei Guo^1,3^

*** Correspondence:** qxb6301@sina.cn; Tel.: +86-373-339-3277

# Supplementary Figures


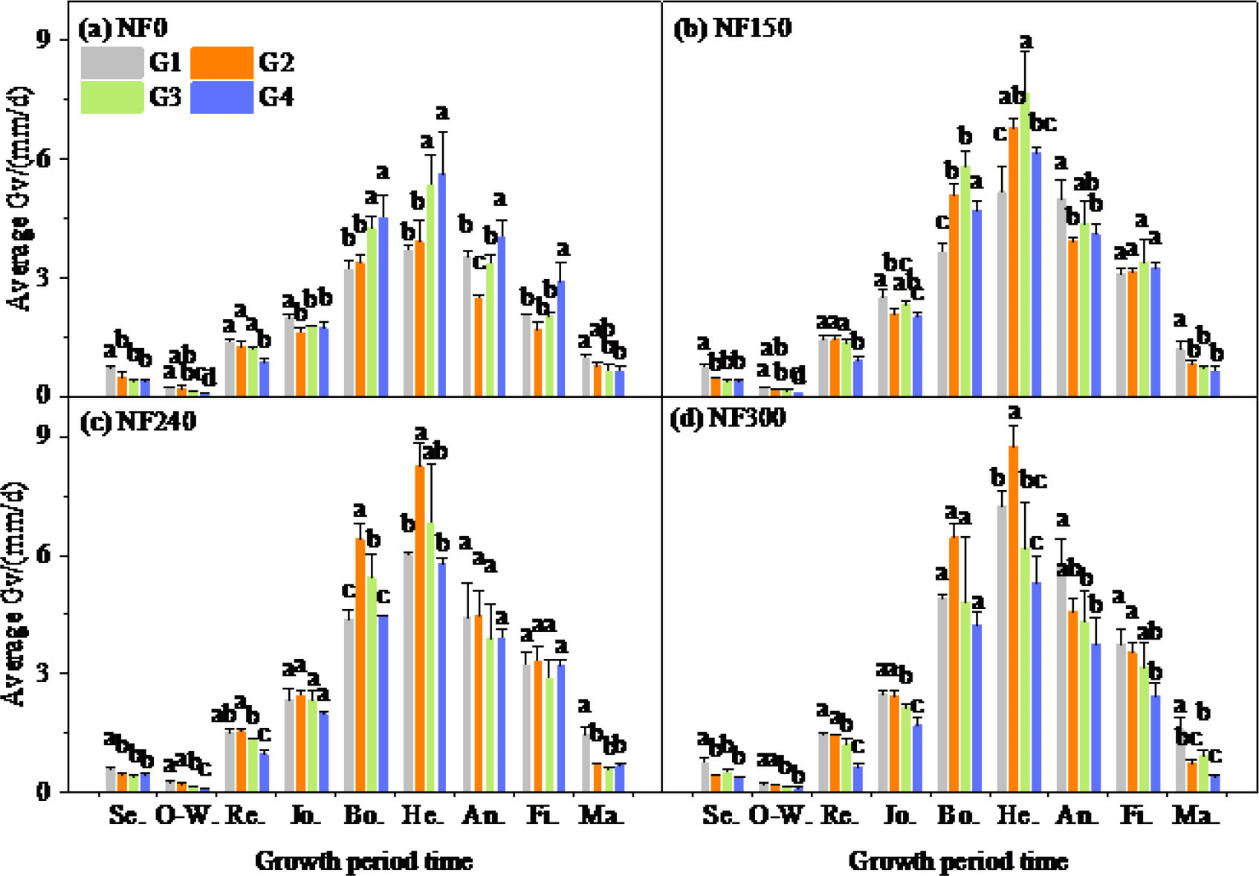


Note: Se., O-W., Re., Jo., Bo., He., An., Fi. and Mat. mean seeding, over-wintering, regreening, jointing, booting, heading, anthesis, filling and maturity stage respectively. According local irrigation habits, winter wheat was irrigated at the beginning of the over-wintering period, and water was added to the Mariotte bottle when the air temperature rose above zero. The amount of water added before the regreening stage was calculated as the groundwater evapotranspiration during the over-wintering stage (O-W.-ETgw), and the average groundwater evapotranspiration velocity during the over-wintering stage was obtained by dividing the O-W.-ETgw by the over-wintering time. Lowercase letters indicate significant differences among different WTD under the same N application rates, P < 0.05.

**Supplementary Figure 1.** Daily groundwater evapotranspiration velocity winter wheat treated with different WTD in N application groups at each growth stage


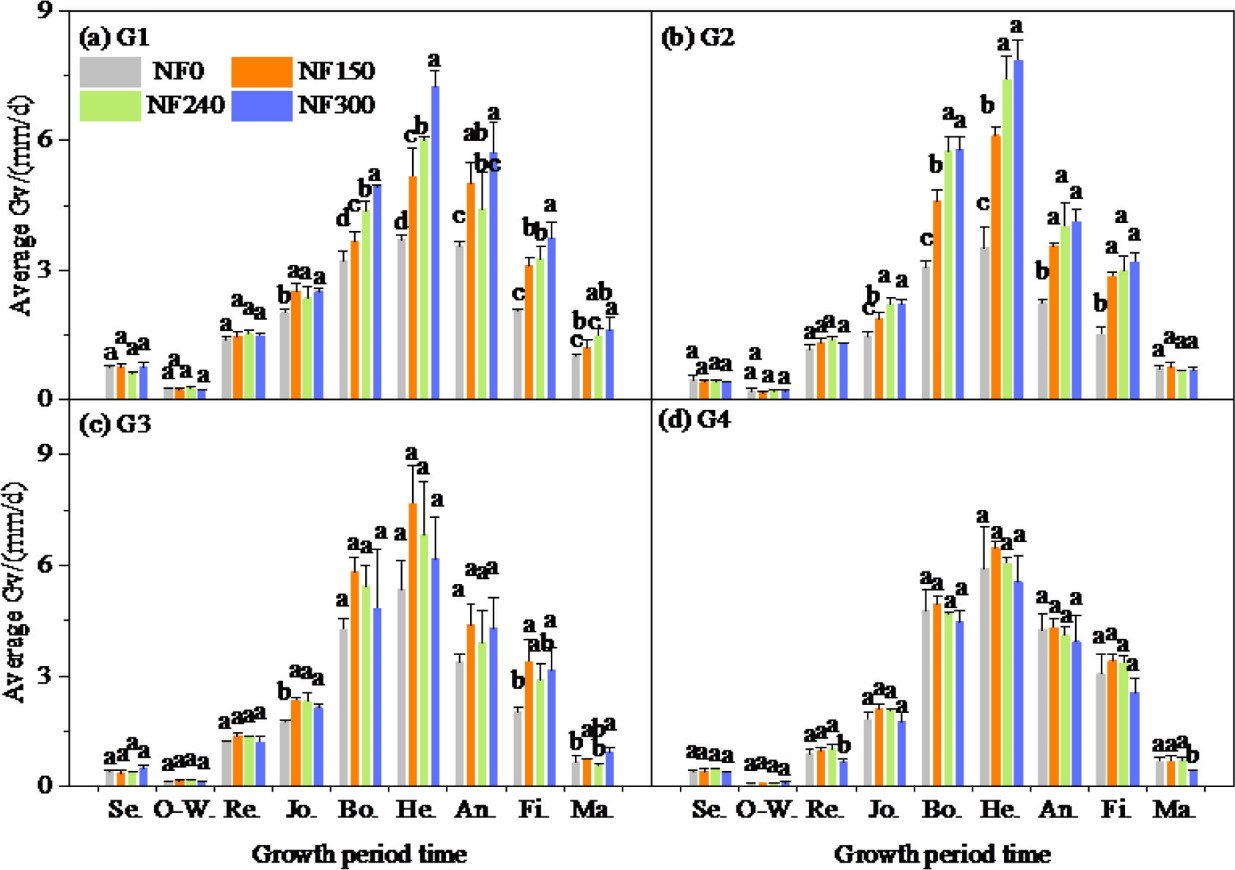


Lowercase letters indicate significant differences among different N application rates under the same WTD, P < 0.05.

**Supplementary Figure 2.** Daily groundwater evapotranspiration velocity winter wheat treated with different N application in WTD groups at each growth stage


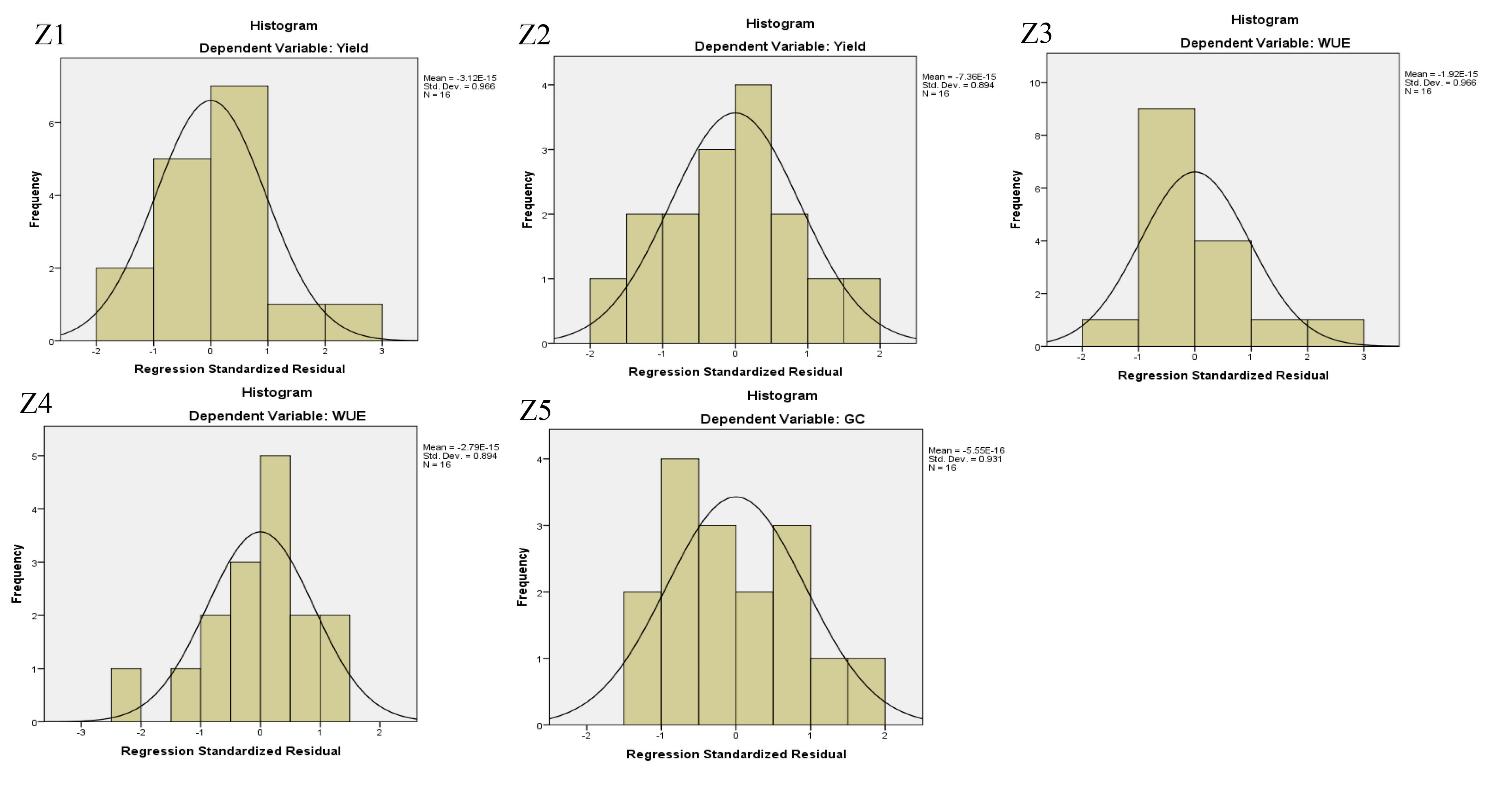


**Supplementary figure 3.** Histogram of regression analysis in the table 4

**
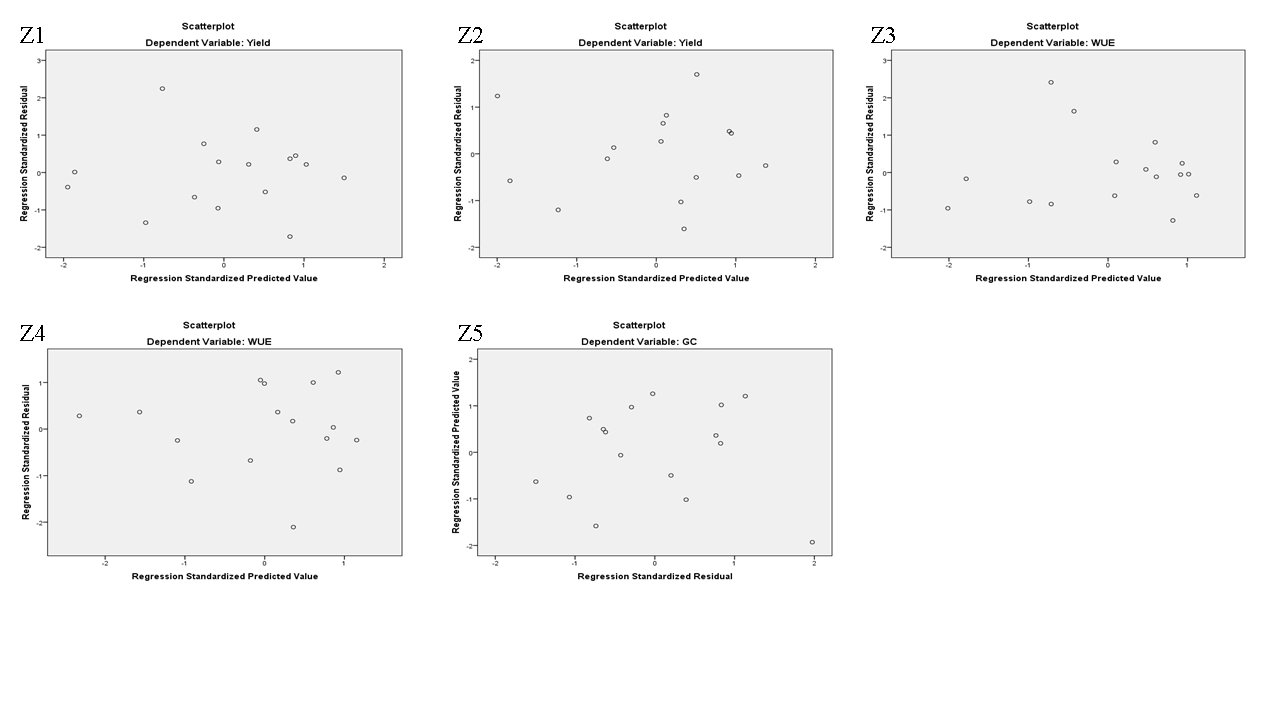
**

**Supplementary figure 4.** Model errors residual scatterplot of regression analysis in the table 4
